# Subviral Dense Bodies of Human Cytomegalovirus Induce an Antiviral Type I Interferon Response

**DOI:** 10.3390/cells11244028

**Published:** 2022-12-13

**Authors:** Inessa Penner, Nicole Büscher, Mario Dejung, Anja Freiwald, Falk Butter, Bodo Plachter

**Affiliations:** 1 Institute for Virology, University Medical Center of the Johannes Gutenberg-University, 55131 Mainz, Germany; 2 Institute for Molecular Biology, 55128 Mainz, Germany

**Keywords:** human cytomegalovirus, subviral particles, dense bodies, interferon-β, interferon-regulated genes, IRGs

## Abstract

(1) Background: Cells infected with the human cytomegalovirus (HCMV) produce subviral particles, termed dense bodies (DBs), both in-vitro and in-vivo. They are released from cells, comparable to infectious virions, and are enclosed by a membrane that resembles the viral envelope and mediates the entry into cells. To date, little is known about how the DB uptake influences the gene expression in target cells. The purpose of this study was to investigate the impact of DBs on cells, in the absence of a viral infection. (2) Methods: Mass spectrometry, immunoblot analyses, siRNA knockdown, and a CRISPR-CAS9 knockout, were used to investigate the changes in cellular gene expression following a DB exposure; (3) Results: A number of interferon-regulated genes (IRGs) were upregulated after the fibroblasts and endothelial cells were exposed to DBs. This upregulation was dependent on the DB entry and mediated by the type I interferon signaling through the JAK-STAT pathway. The induction of IRGs was mediated by the sensing of the DB-introduced DNA by the pattern recognition receptor cGAS. (4) Conclusions: The induction of a strong type I IFN response by DBs is a unique feature of the HCMV infection. The release of DBs may serve as a danger signal and concomitantly contribute to the induction of a strong, antiviral immune response.

## 1. Introduction

The swift induction of intrinsic cellular defense mechanisms is pivotal for the early control of viral infections. Yet, viruses have developed mechanisms to escape that control, in order to allow the subsequent replication. The human cytomegalovirus (HCMV), a member of the *β-herpesviridae*, is one example of a complex virus that devotes large parts of its DNA genome to genes that help to evade cellular control. A considerable body of literature addresses these mechanisms, such as the interaction of HCMV with the interferon (IFN) response, programmed cell death, or autophagy [1,2,3,4,5,6]. In addition to infectious virions, enveloped, non-infectious particles, termed dense bodies (DBs), are produced in infected cells at late stages of the viral replication cycle [7,8,9]. DBs were also detected in infected endothelial cells, in-vivo, showing that the synthesis of these particles is not a cell culture artefact [9]. Due to their immunogenic properties, DBs have been denoted as a promising vaccine candidate [10,11,12,13,14,15,16].

Up until now, the DB synthesis and release have been described only in infected fibroblasts and endothelial cells. As a permissive HCMV infection in the cell culture is restricted to only few cell types and the virus is strictly species-specific, it is unclear if DBs are also produced in other cell types in-vivo, and if they are produced during a latent infection. However, various cell types were found to be permissively infected in-vivo, using an immunohistochemical staining analysis. It is thus likely that other cell types, including epithelial cells, produce DBs [17,18]. The internal structure of DBs mainly consists of viral tegument proteins. The most abundant component among these is the phosphoprotein pp65. DBs are covered by a membrane that contains the major HCMV surface glycoproteins and thus closely resembles the viral envelope. This enables DBs to enter various cell types in a way comparable to that of infectious virions [19].

The HCMV entry into host cells is a complex process that may occur either by the fusion of the viral envelope with the cell membrane at the cell surface (membrane fusion) or by endocytosis. The HCMV exhibits a broad cellular tropism and therefore the entry mechanisms vary according to the HCMV strain and the cell type (reviewed in [20]). The complex HCMV genome encodes at least 25 glycoproteins that assemble in different viral envelope glycoprotein complexes [21,22,23,24,25]. The pentameric complex (PC), comprising the glycoproteins gH/gL assembled with the proteins encoded by the UL128L (UL128-131) locus, is indispensable for the infection of the epithelial cells, endothelial cells (EC), and leukocytes. The trimeric complex (TC), consisting of gH/gL/gO, is required for the infection of all cell types [20]. In 2016, the platelet-derived growth factor-α (PDGFR-α) was identified as the cellular receptor recognized by the TC. The PDGFR-α—TC interaction mediates the viral entry into the fibroblasts [26,27]. Two years later, the cellular receptor neuropilin-2 (Nrp2) was found to interact with the PC. The Nrp2-interaction is used by the virus for entry into the epithelial and endothelial cells via a low pH-dependent endocytosis [28,29]. Additional receptors reported for the epithelial cell entry include a cluster of differentiation 147 (CD147, also known as basigin), a cluster of differentiation 46 (CD46), and the olfactory receptor family 14, subfamily I, member 1 (OR14I1) [29,30,31,32,33]. Although this has not been formally shown, it is reasonable to assume that DBs enter the host cell in the same way as the virions from the parental HCMV strain [34].

Little attention has been given to the impact of DBs on the cell and their role in infections. It was thus reasonable to ask if these particles interfere with the intrinsic cellular defense mechanisms in a way comparable to that of the virus itself.

Interferon regulated genes (IRGs) are induced after the virus infection via the expression and secretion of the type I IFNs. Immediately after the virus entry into the host cell, the intracellular sensors, known as the pattern recognition receptors (PRRs), detect the pathogen-associated molecular patterns (PAMPs) of the invading pathogen and activate an innate immune response to eliminate the pathogen [35]. In mammalian cells, two classes of PRRs can be distinguished: the membrane-bound PRRs, which include Toll-like receptors (TLRs) and C-type lectin receptors (CLRs), and the cytosolic PRRs, which include cytosolic RNA sensors (CRSs), cytosolic DNA sensors (CDSs), and sensors of the inflammasome pathway [35].

**Figure 1:**
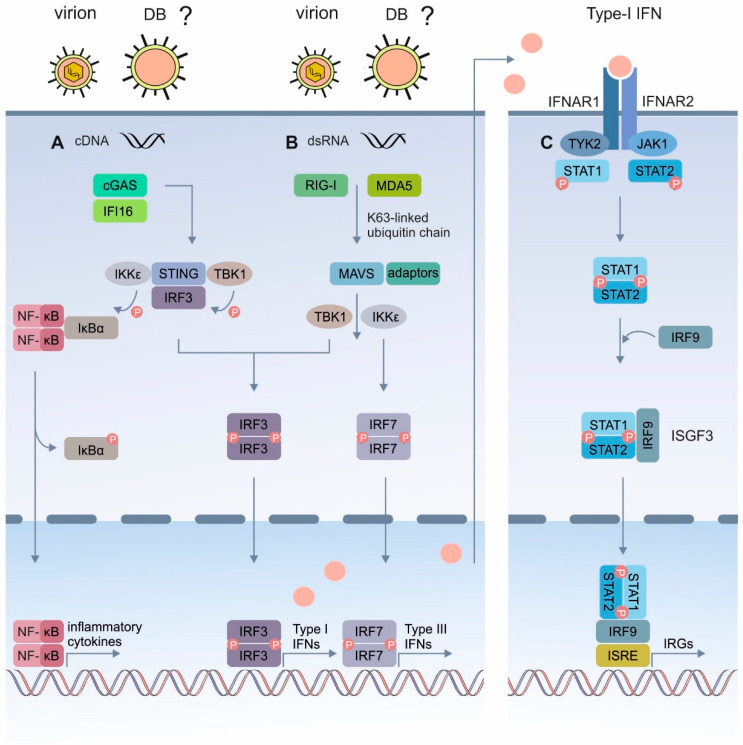
Schematic representation of the IRG induction following the cytosolic sensing of the virus infection. (**A**) Mechanism of the cytosolic DNA-mediated type-I IFN induction. Cytosolic (virus)- DNA is sensed by the cytosolic DNA sensors, such as IFI16 or cGAS. This triggers the STING-TBK1-IRF3 or IKKs- NF-κB signaling cascades. Upon activation, the STING translocates from the ER to the Golgi, where it recruits the kinases TBK1 and IKK, leading to the phosphorylation of IRF3 and IκBα, respectively. Phosphorylated IRF3 dimerizes, translocates to the nucleus, and activates the transcription of the type-I IFN genes. The phosphorylation of IκBα leads to its dissociation from the complex and the translocation of NF-κB to the nucleus, where it activates the transcription of the genes encoding the IFNs and proinflammatory cytokines. (**B**) The RLR signaling pathway. RIG-I or MDA5 are activated by cytosolic (virus)- RNA and interact with MAVS. MAVS becomes associated with several adaptor proteins that lead to the activation of the cytosolic kinases TBK1 and IKKε. Phosphorylation of the transcription factors IRF3 and IRF7 leads to their translocation into the nucleus and the production of type I or type III IFNs. (**C**) Type-I IFN signaling through the JAK/STAT pathway. Binding of type-I IFNs to the heterodimeric receptor IFNAR, which comprises IFNAR1 and IFNAR2, results in the autophosphorylation and activation of the kinases JAK1 and TYK2, which phosphorylate STAT1 and STAT2. The phosphorylated STATs dimerize and associate with IRF9 to form the transcriptional activator complex ISGF3. ISGF3 translocates to the nucleus where it binds to the IRSE element and activates the transcription of the IRGs. DNA, cytosolic DNA; dsRNA, double stranded RNA; RIG-I, retinoic acid-inducible gene; MDA5, melanoma differentiation associated protein 5; MAVS, mitochondrial antiviral signaling protein; IFI16, IFN-inducible protein 16; cGAS, cyclic GMP/AMP synthase; IκBα, inhibitor of the nuclear factor kappa B; IFNAR, interferon- α/β receptor; IKK, inhibitory kappa B kinase; IRF3, interferon regulatory factor 3; IRSE, IFN-stimulated response element; JAK1, Janus kinase 1; NF-κB nuclear factor κappa B; STAT1/2, signal transducer and activator of transcription 1/2; STING, stimulator of interferon genes; TBK1, TANK- binding kinase 1; TYK2, tyrosine kinase 2.

The recognition of the pathogen-derived nucleic acids by the CRSs or CDSs, is a potent trigger of the IFN induction (Figure 1A). Most CDSs seem to activate the IFN response via the key adaptor protein stimulator of the interferon genes (STING; reviewed in [36]). Upon the stimulation by a DNA ligand, the activated STING translocates from the endoplasmic reticulum (ER) to the perinuclear-Golgi region, where it interacts with the tumor necrosis factor receptor-associated factor (TRAF) family member associated NF-kappa B activator (TANK)-binding-kinase-I (TBK1) or IkB kinase (IKK) [37]. Following the phosphorylation by TBK1, the STING recruits the interferon regulatory factor 3 (IRF3), which is also phosphorylated by TBK1. The phosphorylated IRF3 dimerizes and enters the nucleus, where it stimulates the expression of the type I IFN genes (Figure 1A). In parallel, the IkBα phosphorylation by IKK results in the translocation of the nuclear factor kappa-light-chain enhancer of the activated B-cells (NF-κB) to the nucleus and in the corresponding transcriptional expression of the inflammatory cytokines (Figure 1A) [37,38]. The retinoic acid-inducible gene I (RIG-I)-like receptors (RLRs) sense RNA in the cytosol [39]. This family of cytoplasmic PRRs encompasses three members, namely RIG-I, the melanoma differentiation associated protein 5 (MDA5), and the laboratory of genetics and physiology 2 (LGP2). Using the virus infection models, it has been demonstrated that RIG-I and MDA5 are essential for the antiviral defense and the type I IFN induction by RNA-sensing. LGP2 presumably regulates RIG-I and MDA5 [39]. RIG-I and MDA5 recognize different types of RNA, but use a common TLR 7 signaling pathway (Figure 1B) [39]. The RNA binding by RIG-I or MDA5 results in the attachment of unanchored lysine-63 (K63)-linked polyubiquitin chains to the receptors, which promotes their homotetramerization and stabilization (not shown for simplicity). Following the activation, RIG-I or MDA5 interacts with the mitochondrial antiviral signaling (MAVS) adaptor protein on the mitochondrial membrane. MAVS associates with multiple adaptor proteins, including tumor necrosis factors (TNFs) receptor-associated factor-2 (TRAF-2) and TRAF-6, which recruit TRAF-3 and TANK (not shown for simplicity), to trigger the activation of TBK1 and IKKε. TBK1 and IKKε phosphorylate IRF3 and IRF7, which then homodimerize and translocate to the nucleus, to promote the expression of type I and type III IFNs (Figure 1B) [39]. The released type I IFNs (IFNα/β) bind to the cognate IFNα/β receptor (IFNAR), which is expressed on most cell types and activates a signaling cascade through the canonical Janus kinase and signal transducer and activator of the transcription (JAK/STAT) pathway (Figure 1C) [40]. The engagement of the receptor subunits IFNAR1 and IFNAR2 activate the tyrosine kinases JAK1 and tyrosine kinase 2 (Tyk2), which subsequently phosphorylate and activate the signal transducer and activator of the transcription (STAT) 1 and 2 proteins. The activated STAT proteins heterodimerize and recruit the IFN-regulatory factor 9 (IRF9), to form the IFN stimulated gene factor 3 (ISGF3) complex. The ISGF3 transcription factor translocates into the nucleus and induces the expression of the IRGs, which exert antiviral effects (Figure 1C) [37]. Regarding the HCMV infection, several TLRs and CDSs have been described to recognize the HCMV’s PAMPs [38].

One study, performed by Compton et al., identified TLR2 as a cellular sensor that mediated the innate immune responses to HCMV DB [41]. Apart from this, little is known about the impact of DBs on cells. This study aimed at a comprehensive analysis of the impact of HCMV DB on the cell homoeostasis. Multiple proteins were found to be up- or downregulated following the exposure of the culture cells to DBs. Most prominently, many IRGs were found to be induced by DBs.

## 2. Materials and Methods

### 2.1. Cells and Viruses

Human foreskin fibroblasts (HFFs) were propagated, as described before [42]. Endothelial cells (ECs, HEC-LTTs; [43,44]), kindly provided by Christian Sinzger (Institute for Virology, Ulm University Medical Center, Ulm, Germany), were cultured in endothelial cell growth medium (ECGM Kit, C22110, Promocell, Heidelberg, Germany) or endothelial growth medium (EGM BulletKit; Lonza Sales Ltd., Basel, Switzerland), supplemented with 2 μg/mL doxycycline (Sigma-Aldrich, Saint Louis, MO, USA) in culture vessels coated with 0.1% gelatin (Sigma-Aldrich, Saint Louis, MO; PMID: 26345505). The ECs were used from passage 41 to passage 55. Human embryonic kidney 293T cells (HEK293T) were maintained in Dulbecco’s modified Eagle´s medium (DMEM; high Glucose), supplemented with 10% fetal calf serum (FCS) and 1% penicillin/streptomycin (P/S). HFFs were used for most experiments. The primary fibroblasts are the standard culture system to study the HCMV biology in-vitro. In some experiments HEC-LTTs were used.

For the HCMV virus inoculations, supernatant stocks of the endotheliotropic HCMV strain Towne-UL130repΔGFP, expressing the pentameric complex of the membrane proteins gH/gL/UL128-131 (denoted herein as TR-∆GFP [13] and Towne-BAC (TR-BAC; [45])) were used. The virus supernatant stocks were obtained by collecting the supernatant from infected HFF cultures at 7 days post infection (d.p.i.). The supernatants were harvested and precleared from cellular debris by centrifugation at 1475× *g* for 10 min and then stored at −80 °C.

### 2.2. Purification of DBs and the UV-Inactivation

DBs of the HCMV were prepared, as previously described [46]. For this, HFFs were infected with virus supernatant stocks of TR-ΔGFP or TR-BAC, in the presence of 50 nM of Letermovir (LMV). LMV was added to the cell culture at the time of infection and at further 3-day intervals. The DBs were isolated from cell culture supernatants of the infected HFFs showing a complete CPE via the glycerol-tartrate density gradient ultracentrifugation [47]. For the determination of the DB-protein concentrations, the Pierce™ BCA Protein Assay Kit (23225, ThermoFisher Scientific, Darmstadt, Germany) was used, according to the manufacturer’s protocol.

The purified DBs contain residual amounts of infectious virions. Ultraviolet light (UV) irradiation was used as a standard method to prevent the viral gene expression, in order to avoid confounding the effects by the de-novo viral protein expression and genome replication. Briefly, the DBs were thawed and dispersed in a total volume of 120 µL PBS and transferred onto a spot plate. Following irradiation with UV-light for two minutes at a wavelength of 254 nm, 100 µL of the UV-irradiated DB/PBS solution were mixed with culture medium and added to the cells.

### 2.3. Application of the Virus Supernatants and DBs to the Cells

For the infection of 0.5 × 10^6^ the HFF cells in a 10 cm dish, the virus supernatant stocks were diluted in 2 mL 5% MEM and applied to the cells. Following the 1.5 h inoculation, 8 mL of fresh 5% MEM was added and the cells were incubated at 37 °C and 5% CO_2_, for the appropriate time. For the DB-penetration into the HFFs, 20 μg of the UV-inactivated DBs were diluted in 5% MEM, added to 5 × 10^5^ HFFs and incubated at 37 °C and 5% CO_2_.

For infection of the DB-application to the ECs, 6 × 10^5^ cells were seeded on 0.1% gelatin coated 10 cm dishes and maintained in an ECG medium in the absence of doxycycline. The following day, the medium was completely removed, and the cells were washed three times with washing solution A (10 g NaCl; 0.5 g KCl; 0.44 g NaHCO_3_; 1.13 g D-Glucose; filtered sterile and stored at 4 °C). In order to avoid the inhibition of a HCMV infection by heparin contained in the ECG medium, the cells were preincubated in 5% MEM without bFGF (MEM/-bFGF), for at least 30 min. Then the medium was removed. For the DB-adsorption, 20 µg of UV-inactivated DBs were mixed with 5% MEM/-bFGF and applied to the cells for 2 h. Thereafter, fresh ECGM, without doxycycline, was added to the cells. The ECs were propagated at 37 °C and 5% CO_2_.

### 2.4. Plasmids

The plKO plasmids containing the gRNA for the CRISPR/Cas9-mediated knockout cell lines of cGAS, IFI16, MAVS, RIGI, and STAT1, as well as the corresponding envelope and packaging plasmids pMD2.G and psPAX2, were kindly provided by Melanie Brinkmann (Technische Universität Braunschweig, Institut für Genetik, Braunschweig, Germany.)

### 2.5. Antibodies

The following primary antibodies were used: for the detection of the immediate viral early protein 1 and the phosphoprotein 65, the mouse monoclonal antibodies p63-27 (IE1) and 65-33 (pp65) were kindly provided by William Britt (University of Birmingham, Birmingham, AL, USA). Anti-cGAS (1:500, #PA5-56820, ThermoFisher Scientific, Darmstadt, Germany), anti- IFIT3 (1:3000, #PA5-22230, ThermoFisher Scientific, Darmstadt, Germany), anti-IRF3 (SD2062) (1:500, #MA5-32348, ThermoFisher Scientific, Darmstadt, Germany), anti-ISG15 (F-9) (1:500, #sc-166755, Santa Cruz Biotechnology Inc., Dallas, TX, USA), anti-LC3B (D11) XP (1:1000, #3868, Cell Signaling Technology, Frankfurt am Main, Germany), anti-MAVS (20H41L5) (1:500, #703153, ThermoFisher Scientific, Darmstadt, Germany), anti-MX1 (1:1000, #PA5-22101, ThermoFisher Scientific, Darmstadt, Germany), anti-PML (1:100, #ab53773, Abcam, Cambridge, UK), anti-RIG-I (D12) (1:100, #sc-376845, Santa Cruz Biotechnology Inc., Dallas, TX, USA), anti-STAT1 (42H3)(1:500, #9175, Cell Signaling Technology, Frankfurt am Main, Germany), anti-SP100 (1:100, #HPA017384, Atlas Antibodies, Bromma, Sweden), anti-STING (D2P2F) (1:1000, #13647, Cell Signaling Technology, Frankfurt am Main, Germany), and anti-Tubulin-alpha (DM1A) (1:500, #T6199, Sigma-Alrich, Saint Louis, MO, USA).

As secondary antibodies, donkey anti-rabbit Alexa Fluor™ 680 (1:10,000, #A10043, ThermoFisher Scientific, Darmstadt, Germany), donkey anti-rabbit Alexa Fluor™ 488 (2 drops/mL PBS, #R37118, ThermoFisher Scientific, Darmstadt, Germany), goat anti-mouse Alexa Fluor™ 546, 1:500 dilution, (1:500, #A-11003, ThermoFisher Scientific, Darmstadt, Germany), and IRDye^®^ 800 CW donkey anti-mouse (1:15,000, #926-32212, LI-COR Biotechnology, Bad Homburg, Germany) were used.

### 2.6. Reagents and Kits

The transfection reagents Lipofectamine2000 (#11668019) and Lipofectamine RNAiMAX (#13778150) were purchased from ThermoFisher Scientific, Darmstadt, Germany. The recombinant human IFN-β was purchased from PeproTech, Hamburg, Germany (100 U/mL, #300-02BC). The inhibitor of the Janus protein tyrosine kinases (JAKs) JAK Inhibitor I, was purchased from Merck Millipore, Darmstadt, Germany (20 µm/mL, #420099). The Invitrogen™ DNA-free™ DNA Removal Kit (#AM1906) was purchased from ThermoFisher Scientific, Darmstadt, Germany. The High Pure Viral Nucleic Acid Kit (#11858874001) was purchased from Roche Holding, Basel, Switzerland.

### 2.7. Generation of the HFF-Knockout Cells Using CRISPR/Cas9-Mediated Genome Editing

The HFF-Knockout cells were generated, as described by Gonzalez-Perez and co-workers [48]. Briefly, for the lentivirus production, the HEK293T cells were transfected with the envelope and packaging plasmid pMD2 (0.4 µg, #12259 AddGene, Teddington, UK), psPAX2 (1.6 µg, #12260 AddGene, Teddington, UK), and the respective gRNA containg-pLKO5 plasmid (2 µg) [49], using Lipofectamine 2000. The lentivirus was harvested from the culture supernatant 48 h post transfection and diluted 1:2 with MEM and polybrene (final concentration 4 µg/mL). For the transduction, the HFF cells were seeded in 6-well plates (250,000 cells/well) and incubated with the lentivirus-containing medium, including a centrifugation step at 684× *g* and 30 °C, to enhance the viral entry. Then, after 90 min, the cells were transferred into the incubator for further 4 h at 37 °C and 5% CO_2_. Finally, the medium was replenished with fresh HFF medium. The transduced cells were sorted by flow cytometry for the RFP signal, 96 h post transduction. The Cas9-mediated knockout was verified by immunoblotting.

### 2.8. siRNA Knockdown

The following short-interfering RNAs (siRNA) were obtained from Qiagen, Venlo Netherlands. AllStars negative control siRNA (#S103650318), siRNA targeting cGAS: Hs_C6orf150_5 (#S104131687, target sequence CTCGTGCATATTACTTTGTGA), siRNA targeting IRF3: Hs_IRF3_4 (#S102626526, target sequence CAGCCTCGAGTTTGAGAGCTA), siRNA targeting STING: Hs_TMEM173_1 (#S104132170, target sequence CCGCACGGATTTCTCTTGAGA). The HFFs were plated overnight at 2 × 10^5^ cells per well in a 6-well plate, in the absence of antibiotics. The following day, the cells were transfected with 30 µM of control siRNA, siRNA targeting cGAS, IRF3 or STING using Lipofectamine RNAiMAX (#13778150, ThermoFisher Scientific, Darmstadt, Germany), following the manufacturer’s instructions in Opti-MEM (reduced serum medium) (#31985070, ThermoFisher Scientific, Darmstadt, Germany). The transfection medium was removed after 48 h and the DBs were applied for 24 h. The SiRNA- mediated knockdown was verified by immunoblot.

### 2.9. Immunoblot Analysis

The HFFs or ECs were washed once with PBS (HFF) or washing solution A (EC). The trypsinized cells were harvested and pelleted for 5 min at 270× *g*. The cells were then resuspended in PBS, counted, adjusted to 1 × 10^5^ cells/10 µL Laemmli sample buffer and boiled for 10 min at 99 °C. For the western blot analysis, 2 × 10^5^ cells (20 µL) of the total cell lysates were loaded on Bolt™ 10% Bis-Tris gels (NW00105BOX, ThermoFisher Scientific, Darmstadt, Germany). Following the separation by SDS-PAGE and the transfer onto a polyvinylidene difluoride -membrane (PVDF; Immobilon-PSQ, KgaA, IS-EQ00010, Merck, Darmstadt, Germany), the membrane was incubated in Tris-buffered saline (25 mM Tris (pH 7.5), 150 mM NaCl, and 0.2% Tween 20, TBST), containing 5% nonfat dry milk or 5% bovine serum albumin (BSA, #1120180100, Sigma-Aldrich, Saint Louis, MO, USA). The primary antibodies were incubated overnight at 4 °C. Following three washes with 0.2%-TBST for 10 min, the secondary antibodies were applied for 2 h, protected from the light at RT. Following three more washes with 0.2% TBST for 10 min, the images were acquired using the Odyssey Infrared Imager CLx (LI-COR Biotechnology, Bad Homburg, Germany). The quantification was carried out with the corresponding Image Studio™ Lite software.

### 2.10. Extraction of the Viral DNA

The viral DNA was extracted from 20 µg of DBs dissolved in 100 µL PBS using the High Pure Viral Nucleic Acid Kit (#11858874001, Roche Diagnostics, Rotkreuz, Switzerland), according to manufacturer’s instructions. The extracted viral DNA was eluted in 50 µL elution buffer that was provided with the kit.

### 2.11. Quantitative Real-Time Polymerase Chain Reaction (TaqMan qPCR) and Quantification of the Viral DNA

The detection of the HCMV genomes was assessed by a quantitative real-time polymerase chain reaction (qPCR) using the TaqMan^®^ technology [50,51]. Primers CMV-FP 5′-TCATCTACGGGGACACGGAC-3′ and CMV-RP 5′-TGCGCACCAGATCCACG-3′ were used for amplification. The TaqMan^®^ probe 5′-CCACTTTGCCGATGTAACGTTTCTTGCAT-3′ was directed against the HCMV-specific sequence of the HCMV UL54 gene. The DNA standard for the quantification was prepared by serial dilutions of 10^5^ to 10 copies of cosmid pCM1049, which contain the UL54 gene of the HCMV [52]. A no-template control (NTC) was included to monitor the unspecific amplification or contaminations. A real-time PCR was performed in a total reaction volume of 50 µL, containing 5 µL of either the viral DNA sample or the standard DNA solution, 2 units HotStarTaq DNA polymerase (Qiagen, Hilden, Germany), 15 pmol of each primer, and 5 pmol probe. The PCR cycling conditions were 1 min of pre-incubation at 95 °C, 42 amplification cycles of denaturation for 15 s at 95 °C, and annealing for 1 min at 60 °C. The real-time PCR was performed using an ABI Prism 7700 sequence detector (PE Applied Biosystems, Weiterstadt, Germany). All standard dilutions, controls, and DNA samples were run in triplicates. The results were analyzed using the corresponding 7000 System SDS (Sequence Detection Software) software version 1.2.3.

### 2.12. Mass Spectrometry-Based Proteomics

For the mass spectrometry analysis, 0.5 × 10^6^ HFFs were seeded in two 10 cm dishes. Ten μg of UV-irradiated DBs of TR-∆GFP were applied to the cells of each dish. Following 24 h incubation at 37 °C, the cells of both dishes were combined. The 10^6^ cells were lysed in 50 μL 2× Laemmli buffer without bromophenol blue staining and heated at 99 °C for 10 min. Once the samples had cooled down, they were mixed with 1× NuPAGE LDS Sample Buffer (ThermoFisher Scientific, Darmstadt, Germany,) and 100 mM 1,4-Dithiothreit (DTT, #6908.1, Carl Roth GmbH + Co. KG, Karlsruhe, Germany). Finally, the samples were incubated at 70 °C for 10 min and passed to the proteomics core facility (Proteomics Core Facility, IMB Mainz) for the MS measurement. Hereafter, the peptide preparation and processing of the raw data was performed as described in [53]. Briefly, the output table from MaxQuant (version 1.5.2.8) was filtered, including the removal of potential common contaminants and reverse database entries. Only proteins identified with at least two peptides were allowed. Following a second filtering, the proteins with at least one detected ratio in one of the two technical replicates have been included in the analyses.

### 2.13. Statistical Analysis

All statistical tests were performed using the GraphPad Prism version 8.3.0 (538) for Windows (GraphPad Software, San Diego, CA, USA).

## 3. Results

### 3.1. DBs Induce Antiviral IFN-Type I Responses

#### 3.1.1. Mass Spec Analysis Reveals the IRGs to Be Differentially Regulated following the DB Exposure of HFFs

**Figure 2:**
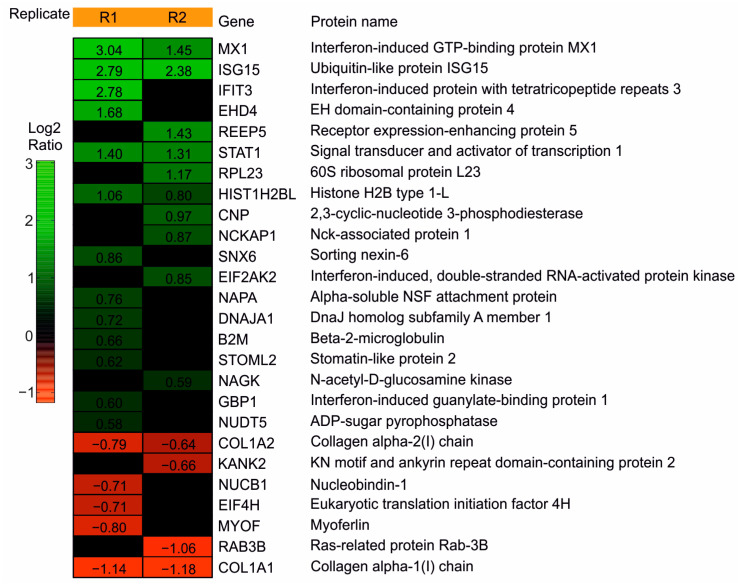
Heatmap of the IRGs regulated in HFFs after the DB incubation. HFFs were exposed to TR-ΔGFP-derived DBs. IRG levels were compared to the levels of the mock cells at 24 h.p.a. The samples were measured on a Q Exactive Plus mass spectrometer. Heatmap of the 26 IRGs that were found to be differentially regulated in HFFs, exposed to 10 μg of DBs. The expression patterns of the proteins were arranged hierarchically, based on the log2 converted normalized ratio of two technical replicates (R1; R2). The log2ratio is represented by a color gradient. Nineteen up-regulated (green) and seven down-regulated (red) IRGs with a fold-change of at least 1.5 (log2ratio ± 0.58) in one of the technical replicates, were considered to be differentially regulated. Black areas represent the values that did not reach the threshold of ±0.58.

To analyze the impact of DBs on the cellular proteome of HFFs, an initial experiment was performed. HFFs were exposed to UV-inactivated DBs or were left untreated. The UV-inactivation was used to avoid the confounding effects by the replication of the residual virions in the DB preparations. The cells were collected at 24 h post application (h.p.a) and processed for MS. A total of 920 proteins were identified in this experiment. Following the filtering, 42 proteins exhibited log2Ratios of at least ± 0.58 (corresponding to a 1.5-fold-change), in at least one of the two technical replicates (R1, R2). These proteins were considered to be differentially regulated (Supplementary Table S1, P1 Up- and downregulated protein groups). Among them, 26 proteins are known to be IFN responsive, according to the INTERFEROME database (http://www.interferome.org/; accessed on 7 August 2020) of the IFN Regulated Genes (IRGs, Supplementary Table S1, P2). The heatmap in Figure 2 shows the IRGs, hierarchically arranged, based on their log2 transformed ratios. At 24 h.p.a, 19 of these IRGs were found to be upregulated and seven proteins were found to be downregulated in the HFFs treated with DBs. An antiviral function has been described for the majority of the upregulated IRGs [54]. To obtain better protein and peptide yields, we analyzed five additional replicates with an optimized proteomics workflow. These analyses confirmed the induction of IRGs by DBs. Together, these experiments provided the basis for a more detailed analysis by the biochemical methods.

#### 3.1.2. Confirmation of the Mass Spectrometry Results by the Immunoblot Analyses

**Figure 3:**
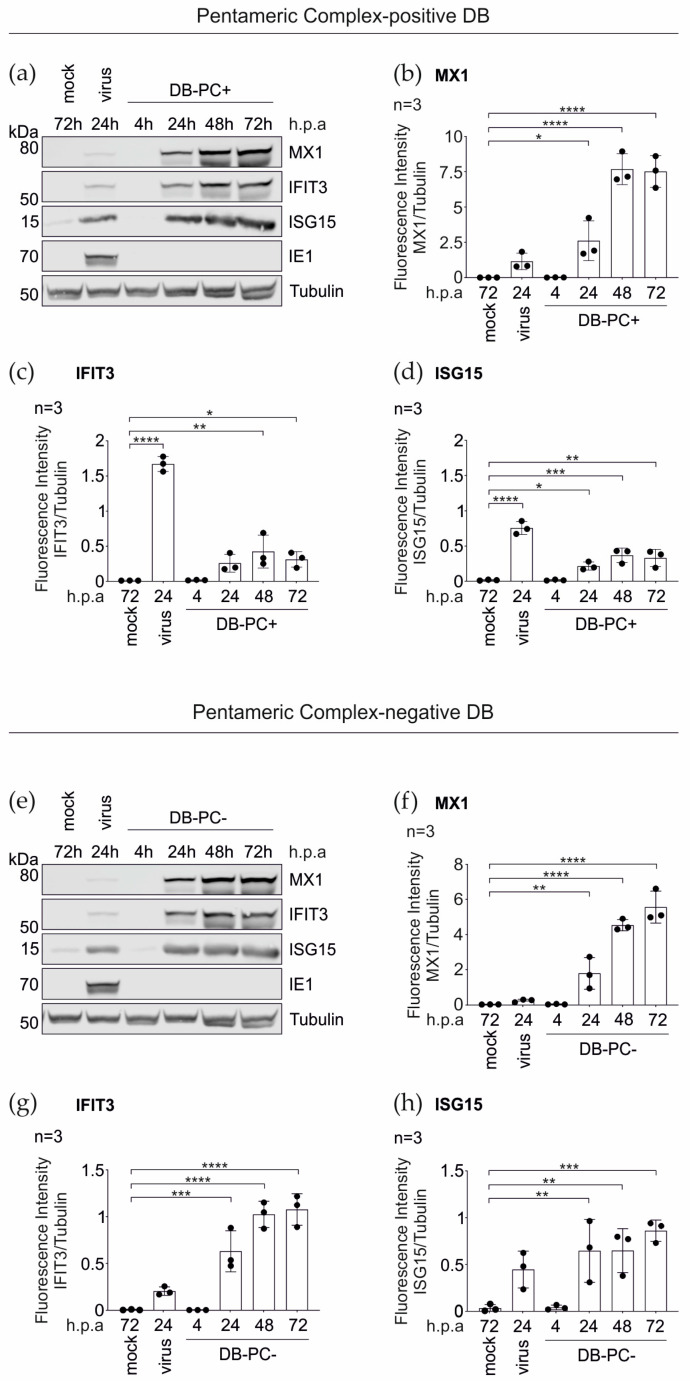
Western blot analysis of the IRG expression in HFFs following the DB-exposure. 5 × 10^5^ HFFs were incubated with 20 μg UV-inactivated DBs of the PC-positive HCMV strain TR-ΔGFP ((**a**–**d**); DB-PC+) or the PC-negative HCMV strain TR-BAC ((**e**–**h**); DB-PC−). Cell lysates were prepared at indicated time points and subjected to SDS-PAGE and immunoblot analyses. Untreated cells (mock) and HCMV infected cells (strain TR-ΔGFP, 100–300 genomes per cell, virus) served as controls. The membranes were probed with antibodies against the IRGs MX1, IFIT3, and ISG15. An antibody against the viral IE1-protein was used to exclude contamination of the DBs with the infectious virus. Tubulin was probed as the sample loading control. For the quantification, the ratio of the measured fluorescence intensity of each IRG protein band to the corresponding tubulin band was calculated using the LICOR Image Studio Lite (Ver 5.2.5) software (**b**–**d**,**f**–**h**). The values of three independent experiments were plotted. Error bars represent the SD. Comparisons between the conditions were calculated using a one-way ANOVA test. The mean of each condition was compared to the mean of the control (mock) condition. * *p* < 0.05; ** *p* < 0.01; *** *p* < 0.001; **** *p* < 0.0001. *n* = Biological replicate.

To validate the MS data, the immunoblot analyses were performed using antibodies directed against the selected IRGs MX1, IFIT3, and ISG15 (Figure 3a–d). The HFFs were stimulated with pentameric complex (PC) -positive DBs (DB-PC+) over a 72 h time course. As a negative control, the cells were left untreated. The infection of HFFs with TR-∆GFP (virus, 100–300 genomes per cell) served as a positive control for the IRG induction. In accordance with the MS data, a robust increase in the expression levels of all three IRGs, upon the DB-treatment, could be confirmed at 24 h (Figure 3a–d). Although the fold-changes were not identical in the immunoblot analyses, compared to the MS data at 24 h.p.a., the tendencies were similar. Interestingly, the maximum induction for all three IRGs was achieved after 48 h of incubation with DBs.

#### 3.1.3. IRG Induction in the HFFs Is Not Dependent on the PC

To determine if the IRGs were also upregulated upon the incubation with DBs that are devoid of the PC, the TR-BAC-derived DBs (DB-PC−) were used and immunoblot analyses were performed, as described before. As shown in Figure 3e–h, the application of DB-PC− also led to an increase of the protein levels of MX1, IFIT3, and ISG15, in a time course of three days.

In conclusion, these data confirm that the DBs elicit the IRG expression in HFFs and show that the induction of IRGs by DBs is independent of the presence of the PC.

#### 3.1.4. DBs Do Not Package STAT1, MX1, IFIT3, or ISG15

**Figure 4:**
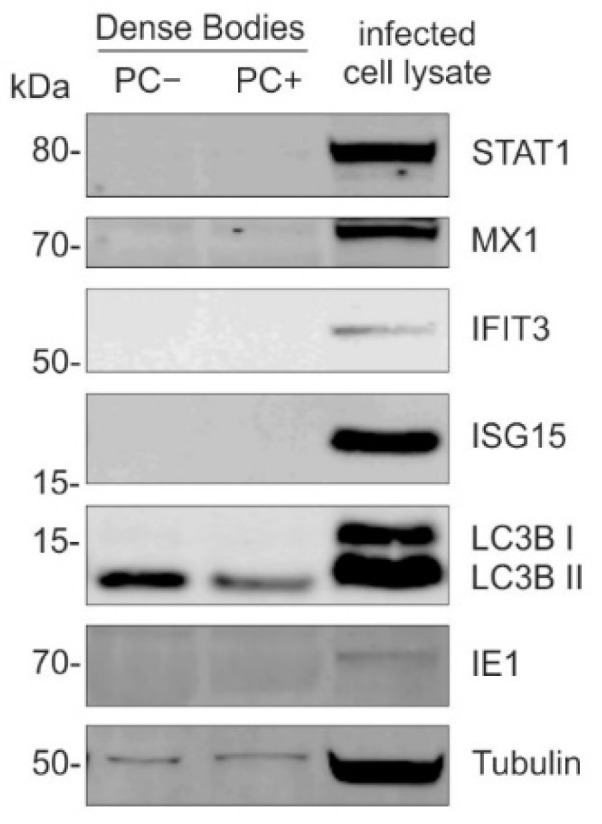
Analysis of the packaging of STAT1 and IRGs MX1, IFIT3, or ISG15 into DBs. Immunoblot analysis of the purified PC-negative and PC-positive DBs. HCMV DBs were gradient purified from the culture supernatants of HFFs infected with the HCMV strain TR-BAC (PC−) or TR-∆GFP (PC+). Thirty µg of each preparation were lysed, subjected to SDS-PAGE, and analyzed by immunoblot, using antibodies against STAT1, MX1, IFIT3, and ISG15. Infected cell lysate (HCMV strain TR-ΔGFP) was subjected as the control. As integral parts of DBs, LC3B and tubulin were visualized and used as the positive control. The detection of the viral IE1 confirmed the infection.

The MS-based studies of the purified HCMV DBs revealed that, in addition to the viral structural proteins, several host cellular proteins, including annexin A2(ANXA2), actin-α-2 and actin-β (ACTN2, ACTB), or the collagen alpha 2 chain (COL1A2) are associated with DBs [22,55]. To exclude the packaging of STAT1, MX1, IFIT3, and ISG15, and the delivery of these proteins into the cells, was accountable for their detection in HFFs, following the DB-application, the immunoblot analyses were performed using purified DBs (Figure 4). HCMV DBs were gradient purified from the culture supernatants of HFFs infected with the HCMV strain TR-BAC (PC−) or TR-∆GFP (PC+). Thirty µg of each preparation were lysed and analyzed by immunoblot using antibodies against STAT1, MX1, IFIT3, and ISG15 (Figure 4). An infected cell lysate (HCMV strain TR-ΔGFP) was subjected as the expression control. As the integral parts of the DBs, LC3B and tubulin were visualized and used as positive controls [5,6,22]. The staining for the IE1 expression confirmed the infection. As shown in Figure 4, neither the PC-negative nor PC-positive, purified DBs contained either STAT1, MX1, IFIT3, or ISG15.

These findings verified that the cellular proteins STAT1, MX1, IFIT3, or ISG15 were not packaged into DBs in the course of their morphogenesis and excluded that the MS analyses on the DB-treated cells were confounded by the introduction of these proteins by incoming particles.

### 3.2. The Pentameric Complex Is Crucial for the DB-Induced IRG Expression in the Endothelial Cells

**Figure 5:**
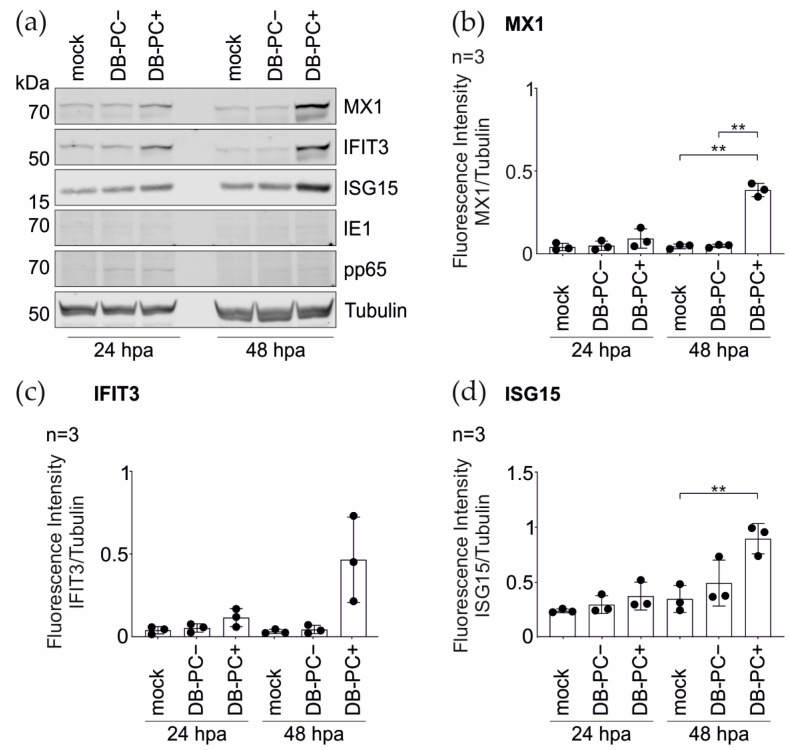
Western blot analysis of the IRG expression in endothelial cells (EC) after DB-exposure. ECs were incubated with 20 μg of UV-inactivated DBs of TR-ΔGFP (DB-PC+) or DBs of TR-BAC (DB-PC−) for 24 h and 48 h. Cell extracts were analyzed for the MX1, IFIT3, and ISG15 expression. (**a**) Western blots were probed with antibodies against MX1, IFIT3, and ISG15. The viral IE1-protein was visualized to verify an infectious virus. Internalization of DBs into endothelial cells was visualized by the detection of the phosphoprotein 65 (pp65). Tubulin was probed as the sample loading control. For quantification in (**b**–**d**), the ratio of the fluorescence intensity of each IRG protein band to the corresponding tubulin band was measured using the LICOR Image Studio Lite (Ver 5.2.5) software. The values of three independent experiments were plotted. Error bars represent the SD. Comparisons between the groups were calculated using the unpaired *t*-test with Welch´s correction. ** *p* < 0.01.

ECs are one predominant target of the HCMV infection, both in-vivo and in-vitro [9,56,57,58]. To analyze whether DBs promote the IRG- induction in ECs, these cells were incubated with TR-∆GFP- derived DBs (DB-PC+). The immunoblot analysis from the total cell lysates prepared at 24 and 48 h.p.a. were evaluated. As shown in Figure 5, the application of DB-PC+ increased the protein levels of MX1, IFIT3, and ISG15 in ECs, compared to the control cells.

Previous work by others had shown that IRGs and inflammatory cytokines are robustly induced following the HCMV application to cells independent of the viral replication [59,60]. Furthermore, cells treated with the HCMV glycoprotein B (gB) strongly induced IRGs [61]. To test if the mere interaction of DBs with the host cell membrane, in the absence of the entry, was already sufficient for the IRG-induction, the ECs were incubated with DBs lacking the PC (Figure 5, DB-PC−). These DBs are unable to enter the EC [62]. The cell lysates were collected and subjected to the immunoblot analysis using antibodies directed against MX1, IFIT3, and ISG15 (Figure 5). No significant increase in the IRG-upregulation was observed in the ECs exposed to DB-PC−, in the course of two days. Altogether, these data indicate that the upregulation of selected IRGs is dependent on the internalization of DBs into the cell.

### 3.3. IRG Induction upon the DB-Application Requires IRF3

**Figure 6:**
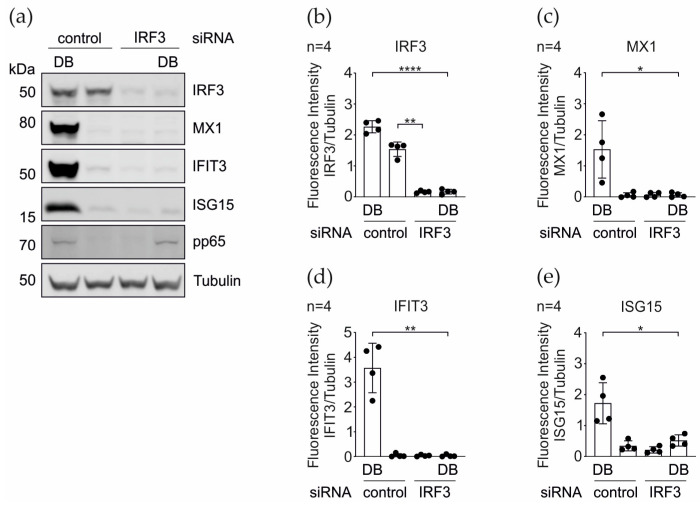
DB-mediated induction of the IRG-expression in the dependence on IRF3. (**a**) Western blot analysis of the IRF3, MX1, IFIT3, and ISG15 protein expression levels in HFFs that were transfected with a siRNA against IRF3 or with a control siRNA and treated with 20 µg of the UV-inactivated TR-ΔGFP DBs. Tubulin levels were used as a loading control and the pp65 levels as the DB internalization control. (**b**–**e**) Quantification of the protein levels in (**a**). The ratio of the measured fluorescence intensity of each protein band to the corresponding tubulin band was calculated. The values of four independent experiments were plotted. Comparisons between groups were calculated using an unpaired *t*-test with Welch´s correction. * *p*< 0.05; ** *p* < 0.01; **** *p* < 0.0001.

In the context of the HCMV infection, Ashley and colleagues demonstrated that the promoters of several core IRGs, including IFIT1, IFIT3, MX1, and ISG15, are transcriptionally induced by the activation of the transcription factor IRF3 and therefore function independent of the IFN responses [60]. To examine whether the IRF3 knockdown interfered with the DB- mediated IRG induction, we silenced the IRF3-expression in HFFs using the siRNA interference. The verification of the IRF3- knockdown was carried out by a western blot analysis. As shown in Figure 6a,b, the expression of IRF3 was efficiently abrogated at the protein level. As expected, the DBs, but not the control siRNA alone, induced the MX1, IFIT3, and ISG15 expression. The knockdown of IRF3 resulted in a complete abrogation of the same IRG expression levels (Figure 6). Thus, the DB-mediated induction of these IRGs is dependent on the IRF3 expression.

### 3.4. Induction of the IRGs by the DBs Depends on the Functionality of the IFN Signaling Pathway

The canonical induction of the IRG transcription is mediated by the activation of the Janus tyrosine kinase/signal transducer and activator of the transcription (JAK/STAT) signaling pathway, initiated by type I, II, and III IFNs [63,64].

**Figure 7:**
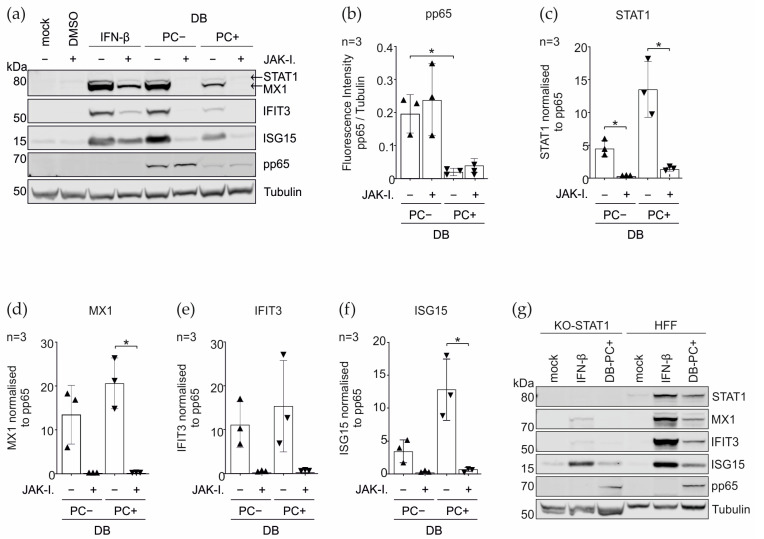
Immunoblot analysis on the relevance of the IFN-signaling pathway for the DB-mediated IRG induction. HFFs were pretreated with either JAK inhibitor I (20 μM/mL) or DMSO. One hour later, the cells were either left untreated (mock), treated with IFN-β (100 U/mL), or treated with 20 μg of the UV inactivated DBs of TR-BAC (PC−) or TR-ΔGFP (PC+). Each sample was further co-treated with JAK inhibitor I for 24 h. (**a**) Representative immunoblot analysis of the STAT1 expression and the downstream IRG protein levels of MX1, IFIT3, and ISG15 in the HFFs upon the JAK inhibitor I treatment, compared to the untreated control cells. IFN-β treatment was used to control for the effects of the inhibitor on the JAK-STAT signaling cascade. Tubulin was used as a protein loading control. Pp65 was used as a DB internalization control. (**b**–**f**) Quantification of the reduction of the IRG protein expression, by calculating the ratio of the fluorescence intensities relative to tubulin and the normalization to pp65. Comparisons between the groups were calculated using an unpaired *t*-test with Welch´s correction. The values of three independent experiments were plotted. * *p* < 0.05. ▲Pentameric complex negative Dense Bodies; ▼ Pentameric complex positive Dense Bodies. (**g**) IFN response in the STAT1 CRISPR/Cas9 knockout HFFs (KO_STAT1). Lysates of the mock-treated, IFN-β-treated (IFN-β, 100 U/mL), and TR-ΔGFP-derived DB (20 µg) treated HFFs were made and analyzed for expression of STAT1, MX1, IFIT3, and ISG15. Additionally, the detection of the tubulin expression was used as a loading control and viral phosphoprotein pp65 as a DB internalization control.

To investigate whether the increased expression of the IRGs STAT1, MX1, IFIT3, and ISG15, upon the incubation with DBs, was dependent on the type I IFN- initiated JAK/STAT- signaling or in an IFN- independent manner, the JAK inhibitor I (JAK-I.) (synonyms: Pyridone6, P6) was used to block the activity of the Janus kinase family enzymes JAK1, JAK2, JAK3, and TYK2, consequently impairing the STAT phosphorylation and the downstream transcription initiation. The effect on the IRG expression was examined by the immunoblot (Figure 7). The HFFs treated with IFN-β (IFN-beta) or the DB induced strong protein expression levels for all four IRGs after 24 h. The blocking of the JAK/STAT pathway with JAK-I. abolished the DB-mediated induction of STAT1, MX1, and IFIT3. The ISG15 expression was reduced to the basal levels. The IRG induction was not completely blocked following the IFN-β treatment, which was likely due to the concentration of the interferon added for the control.

Furthermore, a striking difference in the amount of the pp65 protein between DB-PC− and DB-PC+, was noticed. Although the protein concentration of the purified DBs was determined via the BCA protein assay kit, the staining for pp65 revealed considerable differences in the protein quantity. The tubulin loading control indicated no notable differences. The cell lysates prepared from HFFs and incubated with DB-PC− had an approximately 9.5-times higher amount in the pp65 protein than the lysates of HFFs incubated with DB-PC + DB (Figure 7b). To determine the relevance of this difference, related to the efficiency to enhance the protein expression levels of the IRGs, the IRG expression ratio was normalized to pp65. The calculated values showed that STAT1, MX1, IFIT3, and ISG15, were more potently induced by DBs expressing the PC, compared to the PC-negative DBs (Figure 7c–f).

To substantiate the data from above, we generated the STAT1 knockout HFFs (KO_STAT1) using the CRISPR/Cas9 technology, to block the JAK/STAT cascade. The lysates of the mock-treated, IFN-β-treated, and DB treated HFFs, were made and analyzed for the expression of STAT1, MX1, IFIT3, and ISG15. The western blot in Figure 7g demonstrates that the STAT-1-dependent signaling is essential for the DB-induced IRG upregulation.

These data highlight that both the IRF3 expression and JAK/STAT signaling are required to induce the IRG expression in HFFs upon the DB incubation.

### 3.5. CRISPR Cas9 Knockout and the siRNA Knockdown of PRRs and Their Adaptor Proteins

**Figure 8:**
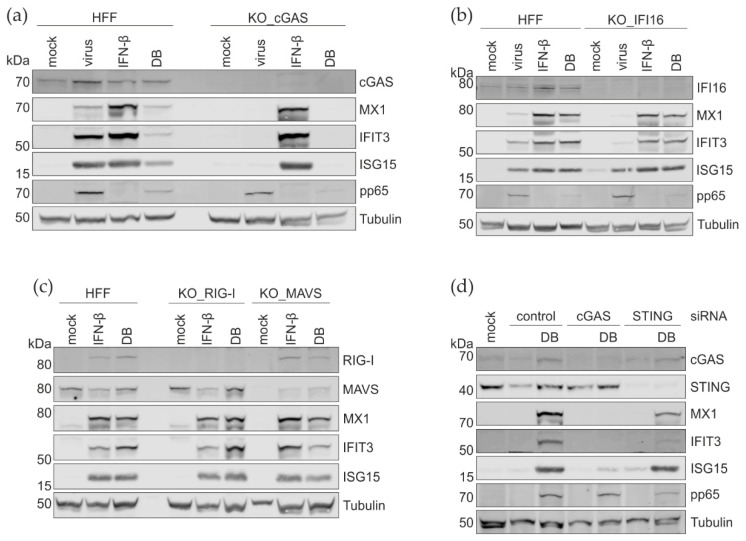
Western blot analysis of the impact of the depletion of the cytosolic sensors on the IRG-induction by DBs. (**a**–**c**) IRG protein levels in cGAS, IFI16, RIG-I, and MAVS CRISPR/Cas9 knockout HFFs. Lysates of the mock-treated, IFN-β-treated (IFN-β, 100 U/mL), infected (virus, TR-ΔGFP), and UV- inactivated DB (20 µg, TR-ΔGFP) treated HFFs were prepared and analyzed for the expression of cGAS, IFI16, RIG-I, MAVS, MX1, IFIT3, and ISG15. (**d**) Western blot analysis of the cGAS, STING, MX1, IFIT3, and ISG15 protein expression levels in HFFs that were transfected with the control siRNA- or siRNA against cGAS and additionally treated with UV- inactivated DBs (20 µg, TR-ΔGFP). Tubulin expression was used as a loading control and viral phosphoprotein pp65 as the DB internalization control.

The invasion of pathogens is sensed by cellular PRRs. To investigate which cellular sensors lead to the production of type I IFNs and the subsequent IRG expression following the DB entry, Cas9-mediated gene editing was used to generate cGAS- and IFI16-, as well as RIG-I- and MAVS- deficient HFF cell lines. The verification of the KO was carried out by a western blot analysis. As shown in Figure 8a–c, the expression of cGAS, IFI16, RIG-I, and MAVS in HFFs was efficiently abrogated at the protein levels. Surprisingly, the DB-mediated induction of the ISG expression was completely abolished only in the KO-cGAS cells (Figure 8a). The abrogation of IFI16, RIG-1, or MAVS had no effect on the ISG levels (Figure 8b,c).

To confirm these results, the siRNA knockdown was used. HFFs were transfected with cGAS- or STING siRNAs (cGAS-KD and STING-KD). cGAS-KD led to the abrogation of the IRG induction by DBs, as expected (Figure 8d). STING-KD was incomplete, consequently leading to a clear but also incomplete reduction of the IRG-induction following the DB-exposure. Taken together, these results confirm that cGAS is required for the IRG activation by DBs, whereas the other PRRs tested appear to be unnecessary for the IRG induction by DBs.

### 3.6. DNase Treatment of DBs Does Not Alter the IRG Expression

It is well known that purified DBs contain DNA [8], thus explaining the cGAS sensing of the DB entry. At this point, it was still unclear if DNA attached to the outer surface of DBs or DNA contained in the internal structure of these particles was mediating the effect. To test whether the DNA that is attached to the surface of DBs was responsible for the cGAS-mediated sensing, 20 µg of TR-∆GFP-DB were treated with DNase for 30 min and subsequently added to HFFs for 24 h. The cell lysates of the mock- and DB- treated cells, in the presence or absence of DNase, were analyzed for the expression of the selected IRGs by western blotting. Surprisingly, the pre-incubation of DBs with DNase had no impact on the IRG expression levels, compared to the control DB. This finding suggested that the DNA that triggers the cGAS- sensing must be located inside the DBs.

**Figure 9:**
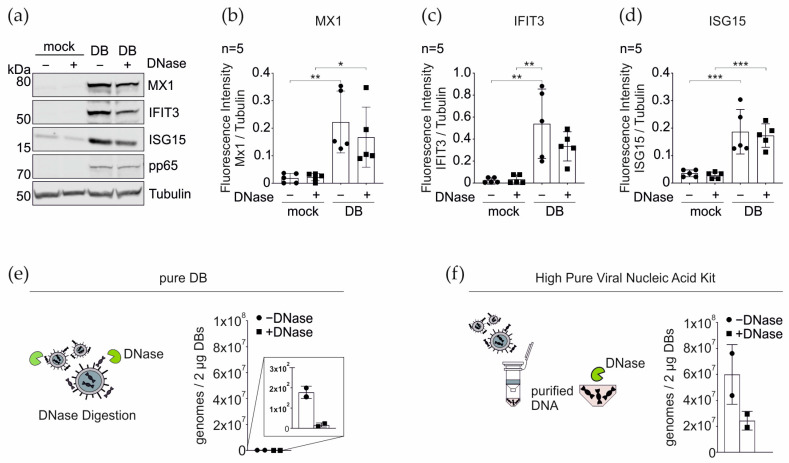
Western blot and qRT-PCR analyses of the impact of the DNase treatment of the purified DBs on the IRG expression and on the DNA detection in the purified DBs. Twenty µg of TR-ΔGFP-DB were treated with DNase for 30 min at 37 °C and subsequently added to HFFs for 24 h. (**a**) Representative Western blot of the cell lysates of the mock and DB-treated cells, in the presence or absence of DNase, were analyzed for the expression of MX1, IFIT3, and ISG15. Tubulin was used as a loading control. DB internalization was monitored by the viral pp65 levels. (**b**–**d**) Quantification of the IRG expression levels in (**a**). The ratio of the fluorescence intensity of each IRG/tubulin band was measured. The values of the five independent experiments were plotted. Comparisons between groups were calculated using the unpaired *t*-test with Welch´s correction for the indicated group, compared with the untreated (mock) group. * *p* < 0.05; ** *p* < 0.01; *** *p* < 0.001. *n* = 5, five biological replicates (**e**) Schematic representation of the DNase digestion of DNA attached to the surface of the pure DBs. Twenty µg of TR-ΔGFP-DB were digested with DNase for 30 min at 37 °C. For the genome determination, 2 µg of DNase-digested and mock-treated DBs were subjected to the qRT-PCR analysis. (**f**) Schematic representation of the DNA extraction from the inside of DBs via the High Pure Viral Nucleic Acid kit. DNA from 20 µg of TR-ΔGFP- DB was extracted and subjected for the DNase digestion. Two µg of DNase-digested and mock-treated DBs were subjected to the qRT-PCR analysis for the genome determination. ● DNase-free sample, ▪ DNase-treated sample. Shown are the values of two biological replicates.

To investigate the amount of viral DNA attached to DBs, the purified particles were directly analyzed by qPCR without the disruption of the DB structure (Figure 9e). The particles treated with DNase were taken along for the control. Roughly 2 × 10^2^ HCMV genome copies per 2 µg DB were found. The DNase treatment, used for the control, almost completely abrogated the detectability of the HCMV DNA.

To investigate the total amount of DNA associated with DBs, DNA was purified from the DBs using the High Pure Viral Nucleic Acid kit, thereby disrupting the DB structure. The samples were either left untreated or subjected to the DNase digestion and then analyzed via the qPCR (Figure 9f). The DNase activity was confirmed by the plasmid digestion and analyzed on an agarose gel. The 6 × 10^7^ genomes per 2ug DBs were detected when the DNA was purified (Figure 9f). The incomplete digestion of DNA was found when the samples were treated with DNase after purification. Taken together, these results show that the sensing of DBs by PRRs, was mediated by DNA contained within the DB structure and that this DNA represents by far the majority of viral DNA associated with DBs.

## 4. Discussion

Cytoplasmic DBs have already been described, shortly after the cultivation of the salivary gland virus, later termed human cytomegalovirus [7,65,66]. They were found to be released from cells as infectious virions, thus allowing their purification from the cell culture supernatants, by either sucrose or by tartrate gradient ultracentrifugation [8,67]. DBs were considered as cell culture artifacts until their discovery in circulating endothelial cells from patients who had undergone renal transplantation [68]. DBs enter cells and deliver their proteinaceous content into the cytoplasm [16,34,69]. These proteins mostly originate from the viral tegument, as characterized by mass spectrometry [22]. Many of these tegument proteins fulfill regulatory functions, such as the pp71 (pUL82), which is a viral transactivator [70,71]. It would thus have been reasonable to assume that DBs support the HCMV replication. In contrast, we have recently found that DBs act in an antiviral, rather than in a proviral fashion (Penner et al., unpublished results). This prompted us to address the impact of DBs on cells in more detail.

Mass spectrometry analyses identified a number of cellular proteins that were either up- or downregulated upon the DB exposure. Surprisingly, many of the upregulated genes were IRGs. This raised the question of how DBs mediated this IRG induction. Recently published data demonstrated that antiviral IRGs were induced in the absence of the IFN-signaling but were dependent on IRF3 [60]. In agreement with this, Boyle et al. found that the interaction of the recombinant gB or virus infection stimulated the IRGs OAS and ISG54 [72]. Simmen and colleagues confirmed and extended this in a comprehensive high-density cDNA microarray approach and showed that the recombinant gB induced a pattern of IRGs indistinguishable from that seen after exposure to the type I recombinant human IFNs [61]. Since HCMV gB does not bind to the type I IFN receptors (IFNARs), the authors hypothesized that the gB binding to an undefined cell surface receptor would induce IRGs by an unknown pathway. In contrast, we find that the DB-mediated IRG induction was dependent on the functionality of the JAK/STAT pathway, indicating that these particles were sensed after their entry into the cells, leading to a type I IFN production and the downstream IRG induction via the interaction of IFN with its cognate IFNARs.

The laboratory strain DBs lack the PC consisting of the membrane proteins gH/gL/pUL128-131 and they are thus unable to enter the endothelial cells [24,57,62]. The failure of the laboratory strain DBs to increase the MX1, IFIT3, and ISG15 levels after treatment of the endothelial cells supports the notion that DBs need to enter cells for the IRG induction. This is consistent with a work by Netterwald and colleagues, who reported that the induction of IRGs was dependent on a post-entry event following the HCMV infection [73]. Our findings also argue against a direct induction of IRGs via the interaction of the gB with a membrane receptor, which should be retained in the absence of the PC. Thus, we found no evidence that the interaction of the HCMV DBs with the cell surface would directly contribute to the induction of IRGs without an IFN induction. This is, however, mostly based on the failure of the PC-negative DBs to induce IRGs in ECs. Since the PC is required for the infection of these cells, it cannot be excluded that the interaction of the PC-negative DBs with the cell membrane was impaired because of the lack of the complex. Additional studies are necessary to evaluate if, e.g., the interaction of the DBs with TLRs may contribute to the interferon response to these particles. On the contrary, the IRG induction was seen in comparable levels in HFFs, irrespective whether PC-positive or PC-negative DBs were used, showing that the PC is not required for the IRG induction in HFFs. Overall, the data suggest that the delivery of the internal content of DBs into the cytoplasm of cells, appears to be a prerequisite for the initiation of the IFN-IRG pathway.

The expression of type I IFNs as a response to the invading pathogens is mediated by several pathways. Major sensing mechanisms depend on the detection of cytoplasmic DNA or double-stranded (ds) RNA through PRRs (see Figure 1). Although cGAS is considered as the main cytosolic and nuclear DNA sensor for the HCMV, it is reasonable to assume that the HCMV is also sensed by cytosolic RNA sensors. Actually, the HCMV encodes several proteins that interfere with the sensors of viral RNA (RIG-I) or the adaptor proteins (MAVS) that trigger antiviral responses, including the production of the type I interferon [74,75,76]). As DBs contain both DNA and RNA [8,77,78], which are delivered into the cytoplasm, it was unclear which the molecules were sensed by the cytosolic PRRs and were responsible for the IRG induction.

Both the siRNA knock-down experiments, as well as the studies on the CRISPR/Cas9 knockout HFFs showed that cGAS is the major DNA sensor for the DB-mediated IRG induction. In contrast, the IFI16 CRISPR/Cas9 knockout HFFs showed no impairment in the IRG induction following the DB incubation, indicating that IFI16 was either not involved in the DB sensing or its sensing function was impaired. Pp65, the major component of DBs, inhibits the IFI16-mediated innate signaling by binding to its pyrin domain [79]. Furthermore, IFI16 primarily acts as a nuclear DNA sensor [80,81]. Recent studies, however, showed that IFI16 shuttles between the nucleus and the cytoplasm upon the cytomegalovirus infection, possibly relocating IFI16 to the site of the DB DNA sensing [82]. The cytoplasmic translocation is, however, mediated through phosphorylation by the viral kinase pUL97 [82,83]. Although pUL97 is found in DBs, the amount may not suffice to mediate the IFI16 phosphorylation and translocation, which is seen primarily at late stages of infection, when pUL97 is expressed to high levels [22]. All this together may explain why the DB-mediated IFN-IRG induction was unimpaired, despite the absence of IFI16.

Previous studies showed that, upon infection, the HCMV tegument proteins pp65 or pp71 interfere with components of the cGAS/STING/IRF3 axis in order to evade the IFN response [84,85,86]. The introduction of these proteins via DBs does not seem to display the same evasive effects (Figure 3). It remains unclear whether pp65 and pp71 execute different functions when introduced into the cell via DBs or if the sensing, mediated by the release of DNA into the cytoplasm, abrogates their interference with cGAS/STING/IRF3. Further studies are needed to investigate this issue.

RNA sensing in the cytoplasm is mediated by RIG-I-like receptors (RLRs). These are cytoplasmic RNA helicases, such as RIG-I and MDA5 (reviewed in [39,87]). Engagement of these receptors with immunostimulatory viral RNAs leads to conformational changes that foster the interaction with MAVS, which relays the signal to the TBK1 to activate IRF3 (see Figure 1). It is well established that the HCMV DBs contain RNA [77,78]. Thus, it was reasonable to assume that DBs might induce the IFN-IRG pathway by RNA sensing. However, exposure of RIG-I and the MAVS CRISPR/Cas9 knockout HFFs to DBs had no impact on the induction of MX1, IFIT3, or ISG15, compared to the control HFFs. One argument for why RNA-sensors were not required for the IRG-induction might be that the amount of RNA in DBs was insufficient. A more likely explanation, however, could be that the RNAs packaged into the DBs were not immunostimulatory. Moreover, the finding that microRNAs are packaged, the nature of the RNA in DBs is unclear. Particularly, it is unknown if dsRNAs are contained in DBs, which induce a strong IFN response. Thus, it remains unclear at this point why there was no RNA-sensing involved in the DB-mediated IFN-IRG induction.

DBs are synthesized both in-vitro and during the natural infection in humans. They are released in large amounts from the HCMV-infected cells. This raises the question about the function of these particles. To some extent, DBs resemble exosomes which are released from cells, in order to mediate cell-cell communication (reviewed in [88,89]). These vesicles carry lipids, proteins, mRNAs, and microRNAs, originating from parental cells. Interestingly, viruses actively manipulate the exosome biogenesis, in order to support the different aspects of their infection, such as morphogenesis and the release of particles, enhancing the infection in neighboring cells, and regulating the viral gene expression (reviewed in [89,90]). Opposed to that, DBs appear to act in an anti- rather than pro-viral fashion, by enhancing the interferon response. One might hypothesize that cells use DBs as a danger signal for neighboring cells, to impede the viral spread in the tissue. In addition, as DBs have been detected in endothelial cells circulating in the blood, a distant effect is conceivable. Of particular interest in this respect are the exceptional antigenic properties of DBs, which are the basis for using these particles for vaccine development [11,13,16,62]. It is conceivable that the release of DBs and their targeting to professional antigen presenting cells, not only supports the induction of the adaptive immune response, based on the remarkable antigenic properties of DBs, and T- and B-cell stimulation. It may also be enhanced by the stimulation of the IFN-response and by generating an inflammatory environment, thus also contributing to an antiviral state in-vivo. Further studies will have to address this issue in further detail.

In conclusion, we have shown here that DBs of the HCMV are unique in that they specifically induce the IFN-IRG pathway, mediated by the DNA sensing through cGAS. This finding will be important for the study of the HCMV-host interactions in cell cultures, as the presence of DBs will have to be considered when interpreting results. In addition, the results will be instrumental in receiving permission for clinical studies in the process of licensing of a DB-based vaccine.

## Supplementary Materials

The following supporting information can be downloaded at: https://www.mdpi.com/article/10.3390/cells11244028/s1, Table S1: Up- and downregulated protein groups.
