# Supplementary material for: Subviral Dense Bodies of Human Cytomegalovirus Induce an Antiviral Type I Interferon Response
Source: Cells. 2022 Dec 13;11(24):4028. doi: 10.3390/cells11244028 (PMC9777239; doi:10.3390/cells11244028)
Supplement: Supplementary file 1 [file cells-11-04028-s001.zip › cells-2026866-supplementary.pdf]

Penner et al., Suppl. Table S1

P1: Up- and downregulated protein groups

Regulated protein groups 24h after DBs exposure

Threshold: log2Ratio 0.58 in minimum one of the 2 replicates
log2Ratio 0.58 = fold increase of 1.5

upregulated
downregulated

						log2.Ratio.DB/mock	
**Protein.ID**	**Protein.name**	**Fasta.header**	**Gene.name**	**Mass (kDa)**	**Razor+ unique peptides**	replicate 1	replicate 2
P20591	Interferon-induced GTP-binding protein Mx1	>sp|P20591|MX1_HUMAN Interferon-induced GTP-binding protein Mx1 OS=Homo sapiens GN=MX1 PE=1 SV=4	**MX1**	75,519	13	3,036731896	1,446187343
P05161	Ubiquitin-like protein ISG15	>sp|P05161|ISG15_HUMAN Ubiquitin-like protein ISG15 OS=Homo sapiens GN=ISG15 PE=1 SV=5	**ISG15**	17,887	5	2,789040599	2,379995477
O14879	Interferon-induced protein with tetratricopeptide repeats 3	>sp|O14879|IFIT3_HUMAN Interferon-induced protein with tetratricopeptide repeats 3 OS=Homo sapiens GN=IFIT3 PE=1 SV=1	**IFIT3**	55,984	3	2,781905211	
Q8N1F7	Nuclear pore complex protein Nup93	>sp|Q8N1F7|NUP93_HUMAN Nuclear pore complex protein Nup93 OS=Homo sapiens OX=9606 GN=NUP93 PE=1 SV=2	**NUP93**	93,381	2		2,220009135
Q9H223	EH domain-containing protein 4	>sp|Q9H223|EHD4_HUMAN EH domain-containing protein 4 OS=Homo sapiens OX=9606 GN=EHD4 PE=1 SV=1	**EHD4**	61,155	2	1,676357687	
Q9UHL4	Dipeptidyl peptidase 2	>sp|Q9UHL4|DPP2_HUMAN Dipeptidyl peptidase 2 OS=Homo sapiens GN=DPP7 PE=1 SV=3	**DPP7**	41,324	3	1,531019572	
Q00765	Receptor expression-enhancing protein 5	>sp|Q00765|REEP5_HUMAN Receptor expression-enhancing protein 5 OS=Homo sapiens OX=9606 GN=REEP5 PE=1 SV=3	**REEP5**	17,758	4	0,076011739	1,426198933
P42224	Signal transducer and activator of transcription 1-alpha/beta	>sp|P42224|STAT1_HUMAN Signal transducer and activator of transcription 1-alpha/beta OS=Homo sapiens GN=STAT1 PE=1 SV=2	**STAT1**	87,334	18	1,398514552	1,311721152
Q92747	Actin-related protein 2/3 complex subunit 1A	>sp|Q92747|ARC1A_HUMAN Actin-related protein 2/3 complex subunit 1A OS=Homo sapiens GN=ARPC1A PE=1 SV=2	**ARPC1A**	41,569	3		1,174719099
P62829	60S ribosomal protein L23	>sp|P62829|RL23_HUMAN 60S ribosomal protein L23 OS=Homo sapiens OX=9606 GN=RPL23 PE=1 SV=1	**RPL23**	8,8994	2		1,174849379
Q99880	Histone H2B type 1-L	>sp|Q99880|H2B1L_HUMAN Histone H2B type 1-L OS=Homo sapiens OX=9606 GN=H2BC13 PE=1 SV=3	**HIST1H2BL**	13,834	4	1,058732098	0,800023053
P09543-2	2,3-cyclic-nucleotide 3-phosphodiesterase	>sp|P09543-2|CN37_HUMAN Isoform CNPI of 2',3'-cyclic-nucleotide 3'-phosphodiesterase OS=Homo sapiens GN=CNP	**CNP**	20,245	2		0,969423787
Q9Y2A7	Nck-associated protein 1	>sp|Q9Y2A7|NCKP1_HUMAN Nck-associated protein 1 OS=Homo sapiens GN=NCKAP1 PE=1 SV=1	**NCKAP1**	128,79	2		0,867936499
Q99733	Nucleosome assembly protein 1-like 4	>sp|Q99733|NP1L4_HUMAN Nucleosome assembly protein 1-like 4 OS=Homo sapiens OX=9606 GN=NAP1L4 PE=1 SV=1	**NAP1L4**	18,152	3	0,021763974	0,867094188
Q9UNH7-2	Sorting nexin-6, N-terminally processed	>sp|Q9UNH7-2|SNX6_HUMAN Isoform 2 of Sorting nexin-6 OS=Homo sapiens OX=9606 GN=SNX6	**SNX6**	33,818	6	0,857025515	0,190684346
P19525-2	Interferon-induced, double-stranded RNA-activated protein kinase	>sp|P19525-2|E2AK2_HUMAN Isoform 2 of Interferon-induced, double-stranded RNA-activated protein kinase OS=Homo sapiens GN=EIF2AK2	**EIF2AK2**	40,68	3		0,848556781
P54920	Alpha-soluble NSF attachment protein	>sp|P54920|SNAA_HUMAN Alpha-soluble NSF attachment protein OS=Homo sapiens OX=9606 GN=NAPA PE=1 SV=3	**NAPA**	9,8571	2	0,755400369	
P31689-2	DnaJ homolog subfamily A member 1	>sp|P31689-2|DNJA1_HUMAN Isoform 2 of DnaJ homolog subfamily A member 1 OS=Homo sapiens GN=DNAJA1	**DNAJA1**	37,044	5	0,719665354	0,562026600
Q71DI3	Histone H3.2	>sp|Q71DI3|H32_HUMAN Histone H3.2 OS=Homo sapiens OX=9606 GN=H3C15 PE=1 SV=3	**H3C15**	10,334	2		0,668226003
P84090	Enhancer of rudimentary homolog	>sp|P84090|ERH_HUMAN Enhancer of rudimentary homolog OS=Homo sapiens GN=ERH PE=1 SV=1	**ERH**	12,259	3	0,658005777	
P61769	Beta-2-microglobulin	>sp|P61769|B2MG_HUMAN Beta-2-microglobulin OS=Homo sapiens GN=B2M PE=1 SV=1	**B2M**	13,946	3	0,657640005	0,318703671
P40121-2	Macrophage-capping protein	>sp|P40121-2|CAPG_HUMAN Isoform 2 of Macrophage-capping protein OS=Homo sapiens OX=9606 GN=CAPG	**CAPG**	36,248	5	0,079156207	0,648371671
Q9UJZ1-2	Stomatin-like protein 2, mitochondrial	>sp|Q9UJZ1-2|STML2_HUMAN Isoform 2 of Stomatin-like protein 2, mitochondrial OS=Homo sapiens OX=9606 GN=STOML2	**STOML2**	33,337	4	0,618896412	0,135887168
Q9UJ70	N-acetyl-D-glucosamine kinase	>sp|Q9UJ70|NAGK_HUMAN N-acetyl-D-glucosamine kinase OS=Homo sapiens GN=NAGK PE=1 SV=4	**NAGK**	36,888	4	0,303108551	0,591266404
P32455	Interferon-induced guanylate-binding protein 1	>sp|P32455|GBP1_HUMAN Guanylate-binding protein 1 OS=Homo sapiens GN=GBP1 PE=1 SV=2	**GBP1**	41,27	3	0,595503661	
Q9UKK9	ADP-sugar pyrophosphatase	>sp|Q9UKK9|NUDT5_HUMAN ADP-sugar pyrophosphatase OS=Homo sapiens GN=NUDT5 PE=1 SV=1	**NUDT5**	25,895	5	0,583037624	0,395245456
P06454-2	Prothymosin alpha	>sp|P06454-2|PTMA_HUMAN Isoform 2 of Prothymosin alpha OS=Homo sapiens OX=9606 GN=PTMA	**PTMA**	11,758	2	-0,586427580	
P46783	40S ribosomal protein S10	>sp|P46783|RS10_HUMAN 40S ribosomal protein S10 OS=Homo sapiens GN=RPS10 PE=1 SV=1	**RPS10**	18,898	5	-0,595423810	-0,522758841
Q9NZN4	EH domain-containing protein 2	>sp|Q9NZN4|EHD2_HUMAN EH domain-containing protein 2 OS=Homo sapiens GN=EHD2 PE=1 SV=2	**EHD2**	61,161	16	-0,602635115	-0,392207499
P20908	Collagen alpha-1(V) chain	>sp|P20908|CO5A1_HUMAN Collagen alpha-1(V) chain OS=Homo sapiens OX=9606 GN=COL5A1 PE=1 SV=3	**COL5A1**	178,51	6	-0,640613772	-0,110363243
P08123	Collagen alpha-2(I) chain	>sp|P08123|CO1A2_HUMAN Collagen alpha-2(I) chain OS=Homo sapiens OX=9606 GN=COL1A2 PE=1 SV=7	**COL1A2**	129,15	24	-0,794007010	-0,641546029
P14406	Cytochrome c oxidase subunit 7A2, mitochondrial	>sp|P14406|CX7A2_HUMAN Cytochrome c oxidase subunit 7A2, mitochondrial OS=Homo sapiens OX=9606 GN=COX7A2 PE=1 SV=1	**COX7A2**	9,4259	2	-0,648281223	
Q63ZY3-3	KN motif and ankyrin repeat domain-containing protein 2	>sp|Q63ZY3-3|KANK2_HUMAN Isoform 3 of KN motif and ankyrin repeat domain-containing protein 2 OS=Homo sapiens GN=KANK2	**KANK2**	90,043	4	-0,057196515	-0,660563586
Q02818	Nucleobindin-1	>sp|Q02818|NUCB1_HUMAN Nucleobindin-1 OS=Homo sapiens OX=9606 GN=NUCB1 PE=1 SV=4	**NUCB1**	51,62	3	-0,709080234	-0,254835883
Q15056-2	Eukaryotic translation initiation factor 4H	>sp|Q15056-2|IF4H_HUMAN Isoform Short of Eukaryotic translation initiation factor 4H OS=Homo sapiens GN=EIF4H	**EIF4H**	25,2	7	-0,709481228	-0,106348320
Q9NZM1-5	Myoferlin	>sp|Q9NZM1-5|MYOF_HUMAN Isoform 5 of Myoferlin OS=Homo sapiens OX=9606 GN=MYOF	**MYOF**	134,71	2	-0,799746768	
Q96FJ2	Dynein light chain 2, cytoplasmic	>sp|Q96FJ2|DYL2_HUMAN Dynein light chain 2, cytoplasmic OS=Homo sapiens GN=DYNLL2 PE=1 SV=1	**DYNLL2**	10,35	2		-0,827168649
Q14192	Four and a half LIM domains protein 2	>sp|Q14192|FHL2_HUMAN Four and a half LIM domains protein 2 OS=Homo sapiens OX=9606 GN=FHL2 PE=1 SV=3	**FHL2**	30,949	5	-0,888033910	-0,246225649
P20337	Ras-related protein Rab-3B	>sp|P20337|RAB3B_HUMAN Ras-related protein Rab-3B OS=Homo sapiens OX=9606 GN=RAB3B PE=1 SV=2	**RAB3B**	24,741	4	-0,186508146	-1,055403920
P55145	Mesencephalic astrocyte-derived neurotrophic factor	>sp|P55145|MANF_HUMAN Mesencephalic astrocyte-derived neurotrophic factor OS=Homo sapiens GN=MANF PE=1 SV=3	**MANF**	21,143	4		-1,173894961
P02452	Collagen alpha-1(I) chain	>sp|P02452|CO1A1_HUMAN Collagen alpha-1(I) chain OS=Homo sapiens GN=COL1A1 PE=1 SV=5	**COL1A1**	138,94	40	-1,137616058	-1,177407578
P04259	Keratin, type II cytoskeletal 6B	>sp|P04259|K2C6B_HUMAN Keratin, type II cytoskeletal 6B OS=Homo sapiens GN=KRT6B PE=1 SV=5	**KRT6B**	60,066	2		-2,436108524
